# Socioeconomic Factors Impact Inpatient Mortality in Pediatric Lymphoma Patients

**DOI:** 10.7759/cureus.624

**Published:** 2016-05-27

**Authors:** Yana Puckett, Anh Ta

**Affiliations:** 1 Surgery, Texas Tech University Health Sciences Center; 2 School of Medicine, Saint Louis University

**Keywords:** pediatric cancer, lymphoma, socioeconomic status, chronic illness, pediatric malignancies

## Abstract

Purpose: Our objective was to determine the risk factors for inpatient mortality of pediatric patients diagnosed with lymphoma through the utilization of a large national pediatric database.

Methods: This cross-sectional study uses data from the Healthcare Cost and Utilization Project Kids' Inpatient Database (HCUP KID) for the year of 2012 to estimate the risk factors for inpatient mortality for pediatric patients diagnosed with lymphoma. All patients diagnosed with lymphoma between the ages of one and 18 years were included. Chi-square test was used to analyze categorical variables. Independent t-test was used to analyze continuous variables.

Results: A total of 2,908 study subjects with lymphoma were analyzed. Of those, 56.1% were male and the average age was three years old. Total inpatient mortality was 1.2% or 34 patients. We found that patients with four or more chronic conditions were much more likely to die while hospitalized (p < 0.0001). In addition, we also saw that patients with median household incomes below $47,999 dollars (p = 0.05) having a need for a major procedure (p = 0.008) were associated with inpatient mortality. Congestive heart failure, renal failure, coagulopathy, metastatic disease, and electrolyte abnormalities were all found to be associated with inpatient mortality.

Conclusions: Pediatric lymphoma mortality in children is not only influenced by their medical condition but also by their socioeconomic condition as well.

## Introduction

Lymphoma is the third most frequent type of cancer in children, making up 11.5% of all childhood malignancies [1]. For children, ages 0-14, approximately 620 cases of non-Hodgkin’s lymphoma (NHL) and 380 cases of Hodgkin’s lymphoma (HL) are diagnosed annually. Over the past 40 years, significant improvements have been made in the diagnosis and treatment of lymphoma. The five-year survival rate of HL has increased from 87% from 1975-1979 to 97% from 2003-2009. Survival rates for NHL increased from 47% to 85% during the same time period [2]. 

Poor survival from lymphoma is strongly associated with a variety of factors. In HL, pretreatment factors that contribute to poor overall survival (OS) include B symptoms, extranodal disease, bulky disease, hemoglobin less than 11.0 g/dL, nodular sclerosis histology, and erythrocyte sedimentation rate of greater than 50 mm/h [3-5]. Poor response to initial therapy contributes to poor survival as well [6]. In NHL, factors that affect survival include response to therapy, stage and site of disease at diagnosis, and genetic abnormalities [7-10]. Central nervous system (CNS) and bone marrow (BM) involvement are strong predictors of outcome. A randomized study of high-risk NHL involving the CNS and BM reported a 30% event-free survival (EFS) in patients who did not respond to initial treatments compared to 97% in those who did.  The same study also reported that patients with BM involvement only and CNS involvement only had a four year EFS of 88% and 82%, respectively. In patients with both CNS and BM involvement, the EFS drops to 61% [7].

The prognosis of lymphoma is also determined by factors that lead to inpatient mortality. Controlling infection is critical in lymphoma management because hematologic cancers are strongly associated with sepsis [11]. Major procedures, including central lines, positive pressure ventilation, and renal replacement therapy, are also linked to higher mortality [11-12]. Lastly, the treatment of lymphoma can cause tumor lysis syndrome, subsequent renal failure, and a significantly poorer prognosis. 

Among these prognostic factors, however, the impact of various comorbidities that are present in children with lymphoma are largely unexplored, despite them being widely researched in adult cancers. While this is due to older patients presenting with more comorbidities, comorbidities are nevertheless strongly associated with lower survival and their presence in children needs to be further explored [13]. 

Furthermore, while there is extensive research on prognosis based on the properties and presentation of lymphoma, there is little research on how non-lymphoma-related factors can affect survival. The most commonly discussed factors are race, sex, and age. The Surveillance, Epidemiology, and End Results Program (SEER) report shows white children having a slightly higher survival than black children in HL and children younger than 10 having a better survival in NHL [14]. Although socioeconomic factors were associated with an increased risk of disease, the study did not report if they had an impact on survival.  A separate study in Switzerland reported an association between paternal education status and lymphoma survival [15]. However, due to the differences in the Swiss economic and healthcare system, there is a need to explore the impact of socioeconomic factors in the United States (US).

## Materials and methods

Utilizing data from the Healthcare Cost and Utilization Project Kids’ Inpatient Database (HCUP KID) compiled for the year 2012, we performed a retrospective analysis of all pediatric lymphoma patients in the United States. Patient-related variables obtained from the database included age (years), sex, race (Caucasian, African American, Hispanic, Asian or Pacific Islander, Native American, and other), in-hospital mortality, and comorbidities associated with each patient. Each record contained discharge diagnosis and procedure codes defined by the 15th International Classification of Diseases, Ninth Revision, Clinical Manifestations (ICD-9-CM).

Comorbid conditions were identified on the basis of both the ICD-9 diagnosis and procedure codes and the Clinical Classification Software diagnosis and procedure classifications. The Statistical Package for the Social Sciences (SPSS) was utilized for data analysis. Chi-square test was used to analyze categorical variables. Independent t-test was used to analyze continuous variables. Estimations with P values less than .05 were considered statistically significant.

Ethical approval was obtained from the Institutional Review Board (IRB) at Saint Louis University prior to the start of the study. Informed consent was obtained at the time of initial treatment.

## Results

A total of 2,908 study subjects with lymphoma were analyzed. Of those, 56.1% were male and the average age was three years. Caucasians comprised 50.9% of the population, followed by Hispanics (18.8%), and African-Americans (12.5%). The average length of stay was 6.16 days. The average patient had a total of approximately two procedures and three chronic conditions. The most common expected payer was private insurance with Medicaid being second. Total inpatient mortality was 1.2% or 34 patients (Table 1).

**Table 1:** Demographics of Study Population (n=2908) SD: Standard Deviation; n: Population Number; LOS: Length of Stay; USD: United States Dollars

Demographics of Study Population (n=2908)	
Gender (Male)	56.1%
Mean Age (SD)	2.97 (1.1)
Mean LOS Days (SD)	6.16 (10.0)
Mean Number of Procedures (SD)	1.7 (2.4)
Mean Number of Chronic Conditions (SD)	3.2 (2.0)
Mortality	1.2% (34)
Primary Expected Payer	
Medicare	0.9% (27)
Medicaid	35.4% (1,028)
Private Insurance	54.6% (1,587)
Self-Pay	2.9% (84)
No Charge	5.9% (173)
Race	
White	50.9% (1,481)
Black	12.5% (364)
Hispanic	18.8% (546)
Asian or Pacific Islander	3.7% (108)
Native American	0.3% (9)
Other	4.7% (136)
Region of Hospital	
Northeast	19.3% (561)
Midwest	23.0% (670)
South	35.6% (1,036)
West	22.0% (641)
Median Household Income (USD)	
1 - 38,999	25.3% (735)
39,000 - 47,999	23.7% (690)
48,000 - 62,999	22.7% (659)
63,000+	26.2% (761)
Most Common Procedure Type	
Blood Transfusion	19.2% (557)
Enteral and Parenteral Nutrition	3.5% (103)
Cancer Chemotherapy	4.8% (141)
Other	6.9% (200)
Nontherapeutic Skin Procedures	2.7% (78)
Diagnostic Spinal Tap	1.4% (42)
Epidural/Spinal Catheter Insertion	1.0% (28)
Central Line Catheter Placement	2.0% (57)
Bone Marrow Transplant	1.0% (30)
Respiratory Intubation/Mechanical Ventilation	1.1% (32)

In performing a comparison of patient demographics in pediatric lymphoma patients in terms of inpatient mortality, patients with four or more chronic conditions were much more likely to die while hospitalized (p < 0.0001). In addition, we also saw that patients with median household incomes below $47,999 dollars were more predisposed to inpatient mortality compared to those making $48,000 and above (p = 0.05). The need for a major procedure was associated with inpatient mortality as well (p = 0.008) (Table 2). There were no statistically significant associations found between gender, race, primary expected payer, and region of the hospital.

**Table 2:** Comparison of Patient Demographics in Pediatric Lymphoma Patients in the United States with In-Hospital Mortality Being the Outcome, 2012 (n=2,908) Chi-Square: Categorical Variables Independent Samples T-Test: Continuous Variables n: Population Number

	Mortality % (n=34)	No Mortality % (n=2,801)	P-Value
Gender (Female)	52.9% (18)	44.9% (1,258)	0.185
Race			
African-American	44.1% (15)	52.3% (1,466)	0.208
Caucasian	23.5% (8)	12.7% (356)	
Hispanic	14.7% (5)	19.3% (541)	
Other	17.6% (6)	3.4% (106)	
Primary Expected Payer			
Medicare	5.9% (2)	0.01% (25)	0.045
Medicaid	32.4% (11)	36.3% (1,017)	
Private Insurance	50.0% (17)	56.1% (1,570)	
Self-Pay	2.9% (1)	3.0% (83)	
No Charge	8.8% (3)	6.1% (170)	
Region of Hospital			
Northeast	17.7% (6)	(555)	0.885
Midwest	23.5% (8)	(662)	
South	41.2% (14)	(1,022)	
West	17.7% (6)	(635)	
Number of Chronic Conditions			
1	5.9% (2)	19.8% (441)	< 0.001
2	0.0% (0)	28.8% (808)	
3	11.8% (4)	24.1% (675)	
4	20.6% (7)	15.0% (421)	
5+	61.8% (21)	18.9% (529)	
Median Household Income			
1 - 38,999	35.3% (12)	25.8% (723)	0.05
39,000 - 47,999	38.2% (13)	24.2% (677)	
48,000 - 62,999	11.8% (4)	23.4% (655)	
63,000+	14.7% (5)	27.1% (759)	
Need for Major Procedure	76.5% (26)	45.3% (1268)	0.008

A comparison of comorbidities in pediatric lymphoma patients was performed as well. A total of 25 comorbidities were analyzed. Of those, congestive heart failure (p = 0.001), coagulopathy (p < 0.0001), fluid and electrolyte disorders (p < 0.0001), metastatic cancer (p = 0.024), neurological disorder (p = 0.027), and renal failure (p = 0.006) were found to be statistically significant in terms of being a risk factor for inpatient mortality (Table 3).

**Table 3:** Comparison of Comorbidities in Pediatric Lymphoma Patients in the United States with In-Hospital Mortality Being the Outcome, 2012 (n=2,908) Chi-Square: Categorical Variables Independent Samples T-Test: Continuous Variables N/A: Not Applicable n: Population Number

	Mortality % (n=34)	No Mortality % (n=2,801)	P-Value
Obesity	0.0% (0)	2.6% (73)	N/A
Deficiency Anemias	14% (5)	9.1% (254)	0.180
Rheumatoid Arthritis/Collagen Vascular Disease	0.0% (0)	0.4% (11)	N/A
Chronic Blood Loss Anemia	0.0% (0)	0.5% (14)	N/A
Congestive Heart Failure	8.8% (3)	0.4% (12)	0.001
Chronic Pulmonary Disease	2.9% (1)	8.2% (229)	0.236
Coagulopathy	41% (14)	11% (314)	< 0.001
Depression	5.9% (2)	4.8% (135)	0.481
Diabetes, Uncomplicated	2.9% (1)	0.9% (26)	0.273
Diabetes, with chronic complications	0.0% (0)	0.1 % (2)	N/A
Drug Abuse	2.9% (1)	1.7% (48)	0.441
Hypertension (combined uncomplicated and complicated)	12% (4)	6.2% (174)	0.151
Hypothyroidism	0.0% (0)	2.1% (59)	N/A
Liver Disease	5.9% (2)	1.4% (39)	.082
Fluid and Electrolyte Disorders	76% (26)	19% (540)	< 0.001
Metastatic Cancer	5.9% (2)	0.7% (19)	0.024
Neurological Disorder	12% (4)	3.4% (95)	0.027
Paralysis	5.9% (2)	1.2% (35)	0.069
Peripheral Vascular Disorders	2.9% (1)	0.2% (5)	0.068
Psychosis	0.0% (0)	1.3% (37)	N/A
Pulmonary Circulation Disorders	2.9% (1)	0.7% (19)	0.210
Renal Failure	12% (4)	2.1% (59)	0.006
Solid Tumor without Metastasis	0.0% (0)	0.6% (18)	N/A
Valvular Disease	2.9% (1)	0.4% (11)	0.132
Weight Loss	8.8% (3)	0.4% (11)	0.160

## Discussion

### Main findings of the study

In this study, household income, the need for a major procedure, the number of chronic conditions, and the number of comorbidities were identified as important risk factors for inpatient hospital mortality in pediatric lymphoma patients.

Children in families with a median income of less than $47,999 had a 1.4 times higher mortality risk while hospitalized than children in families of higher income. This income is lower than the median income in the US as reported by the US Census Bureau [16]. This is consistent with previous analysis between lower socioeconomic status and HL survival and also with socioeconomic studies in adults with diffuse large B-cell lymphoma [17-18].

However, we also found that there is no significany difference in inpatient mortality among different expected primary payers. This result is different from a previous study that suggests uninsured children have a higher inpatient mortality overall. The same study suggests that poorer outcomes may be due to uninsured families depending on emergency services as their source of care and children having more advanced stages when they present [19]. Our data does not indicate that type of insurance affects inpatient mortality in pediatric lymphoma patients. There was no statistically significant difference in mortality between lymphoma patients on medicaid, medicare, private insurance, or self-pay status. Future research needs to be done to see if patients with lower socioeconomic status have more advanced disease when they present. Other socioeconomic factors facing families with lower incomes may be involved. For example, in a high-income country like Switzerland, where there is an above-average educational system and universal health care, lower education status of the father is still associated with a lower lymphoma survival [15]. Therefore, other socioeconomic factors need to be assessed and considered.

To the best of our knowledge, there are no previous studies investigating causes of inpatient mortality in pediatric lymphoma patients. Several studies report the most common cause of death in lymphoma to be infection, renal failure, and hemorrhage [11-12, 20-22]. Our findings echo these studies as renal failure and coagulation abnormalities were significantly associated with inpatient mortality in pediatric lymphoma patients.

Hematologic cancers are more likely to be associated with sepsis and viral infection, which is associated with a higher mortality. This problem is further worsened by both malignancy and treatments causing immunosuppression [11]. Early mortality in advanced mature B-cell malignancies has been observed with extensive surgical procedures, although the result was not statistically significant [22]. Our study found that patients who had the need for a major procedure were much more likely to have an inpatient mortality. However, the top three procedures cited as “major” in the database for those with inpatient mortality were mechanical intubation (32.4%), blood transfusion (14.7%), and enteral or parenteral nutrition (11.8%). These results can generally be explained by these procedures being used only for patients who are already in worse conditions.

In addition, pediatric cancer patients have a higher risk of hospital-associated infections compared to other pediatric patients, especially with central lines [12]. The need for prolonged parenteral nutrition further predisposes these pediatric cancer patients to central line infections [23]. Hematologic cancer patients are also more likely to receive noninvasive positive pressure ventilation and renal replacement therapy in the PICU compared to other cancers and equally likely to use invasive positive pressure ventilation. Each intervention is associated with higher mortality in the PICU [11-12]. Lymphoma patients also commonly need blood transfusions, which can have immunosuppressive effects, putting the patient at risk of infection or viral reactivation [24]. The need for a procedure needs to be carefully assessed, especially in immunocompromised lymphoma patients, so that children are not put at risk of infection and inpatient death.

Having more than four chronic illnesses is a significant risk factor for inpatient mortality, consistent with past studies in adults [13]. Pediatric cancer patients who present with organ involvement and failure had a higher frequency of death [12]. Specific comorbidities associated with increased inpatient mortality were congestive heart failure, coagulopathy, fluid and electrolyte disorder, metastatic disorder, neurological disorder, and renal failure. Higher Pediatric Risk of Mortality 3 (PRISM3) score based on cardiovascular, neurologic, acid-base chemistry, and hematologic derangements were associated with a higher risk of PICU mortality [11]. Metastatic disorder, extranodal disease, and bulky disease are also associated with a worse prognosis in lymphoma, a result also observed in our study [3]. Coagulopathy and neurological disorders being risk factors are supported by BM and CNS involvement in lymphoma, leading to lower survival independently and an even lower survival when patients have involvement of both systems [7]. This suggests inpatient mortality and long-term survival can be improved by effective screening to catch disease before extranodal and metastatic spread, especially to the CNS and BM.

Fluid and electrolyte disorders and renal failure are a common result of tumor lysis syndrome, a frequent complication and risk of mortality in lymphoma [25]. Tumor lysis syndrome typically occurs after the initiation of chemo or radiotherapy and causes hyperphosphatemia, hypocalcemia, hyperkalemia, hyperuricemia, and ultimately acute renal failure [26].  With renal failure being one of the most common causes of death in lymphoma patients, careful monitoring, prevention, and treatment of renal dysfunction is essential in preventing inpatient mortality in lymphoma [20, 27].

### Limitations of the study

This study has a number of limitations. We are unable to collect other socioeconomic factors that may affect the outcome. As shown by previous studies, other factors, such as educational status and compliance, may affect survival [15, 18]. However, if we expanded our data collection to include more variables, there are factors that may not be easily collected in a patient’s chart retrospectively. This can include the parents’ occupation, community support, and other factors that contribute to low socioeconomic status.

We are also limited by the lymphoma-related data provided by the Healthcare Cost and Utilization ProjectKids' Inpatient Database. From this database, we are unable to determine the different types of lymphoma and if the comorbidities are independent of the lymphoma or are results of the lymphoma. We also do not know from the database if the comorbidities were also diagnosed at the time of lymphoma diagnosis or if they were chronic conditions the child was already known to have. Therefore, we cannot know if the increase in mortality is due to the lymphoma disrupting the organ system or due to the existing dysfunction of that system exacerbating the lymphoma. This relationship needs to be further explored to determine how to monitor and manage comorbidities and improve outcomes. Furthermore, our database does not have long-term outcomes and survival, and we are unable to determine if the factors that contribute to inpatient mortality have an effect on long-term survival. In addition, factors, such as expected primary payer and comorbidities that are not identified as risk factors for inpatient mortality, may have an impact on long-term survival. Since many past studies of mortality have been on pediatric cancers in general rather than lymphoma, more studies need to be done to see if factors that affect pediatric cancer mortality affect lymphomas more specifically.

Lastly, this is a retrospective study, and thus, we are unable to determine how the comorbidities were managed and whether more effective management of the specific risk factors led to better survival. This relationship can be explored in future studies to develop better plans to manage pediatric lymphoma patients with comorbidities. 

## Conclusions

In this national children’s database study, we found that pediatric lymphoma mortality in children is not only influenced by their medical condition but also by their socioeconomic condition as well. Medical conditions that contribute to inpatient mortality include comorbidities, the need for a major procedure, and metastatic and extranodal disease. Patients with low median family income are found to have higher mortality. One possible explanation for this result is that patients with low family income may have both higher number comorbidities and a more progressed disease at diagnosis, leading to a higher rate of mortality. We also found no significant difference in mortality among different expected primary payers, indicating that it is not enough for families to be insured. Other factors may exist including parent education level and access to care that contribute to a delay in diagnosis.  Future research needs to be done to see if patients with a lower socioeconomic status present with more advanced disease and what factors contribute to a delay in diagnosis. 
